# Network‐based feature selection reveals substructures of gene modules responding to salt stress in rice

**DOI:** 10.1002/pld3.154

**Published:** 2019-08-12

**Authors:** Qian Du, Malachy Campbell, Huihui Yu, Kan Liu, Harkamal Walia, Qi Zhang, Chi Zhang

**Affiliations:** ^1^ School of Biological Sciences Center for Plant Science and Innovation University of Nebraska Lincoln NE; ^2^ Department of Agronomy and Horticulture Center for Plant Science and Innovation University of Nebraska Lincoln NE; ^3^ Department of Animal and Poultry Sciences Virginia Polytechnic Institute and State University Blacksburg VA; ^4^ Department of Statistics University of Nebraska Lincoln NE

**Keywords:** co‐expression network, data integration, gene modules, LASSO regression, linkage between genome to phenotype

## Abstract

Rice, an important food resource, is highly sensitive to salt stress, which is directly related to food security. Although many studies have identified physiological mechanisms that confer tolerance to the osmotic effects of salinity, the link between rice genotype and salt tolerance is not very clear yet. Association of gene co‐expression network and rice phenotypic data under stress has penitential to identify stress‐responsive genes, but there is no standard method to associate stress phenotype with gene co‐expression network. A novel method for integration of gene co‐expression network and stress phenotype data was developed to conduct a system analysis to link genotype to phenotype. We applied a LASSO‐based method to the gene co‐expression network of rice with salt stress to discover key genes and their interactions for salt tolerance‐related phenotypes. Submodules in gene modules identified from the co‐expression network were selected by the LASSO regression, which establishes a linear relationship between gene expression profiles and physiological responses, that is, sodium/potassium condenses under salt stress. Genes in these submodules have functions related to ion transport, osmotic adjustment, and oxidative tolerance. We argued that these genes in submodules are biologically meaningful and useful for studies on rice salt tolerance. This method can be applied to other studies to efficiently and reliably integrate co‐expression network and phenotypic data.

## 1 INTRODUCTION

Rice (*Oryza sativa*) is arguably the most important crop worldwide. Approximately 3.5 billion people globally rely on the cultivation and distribution of rice for food and economic security. Given its economic importance, considerable efforts are continually made to maximize productivity. However, environmental factors such as drought, salinity, high heat, and submergence are major constraints. Especially, rice is highly sensitive to salt stress (Flowers & Colmer, 2015). This sensitivity is driven by the osmotic effects of excessive Na^+^ in the soil–plant relations and the toxic effects of Na^+^. Therefore, study on salt tolerance in rice is important for food security. Although many studies have identified physiological mechanisms that confer tolerance to the osmotic effects of salinity and documented several mechanisms to limit the toxic effects of Na^+^ on plant growth, the link between rice genotype and salt tolerance is not very clear yet, because salt tolerance is a complex quantitative trait, which involves numerous changes in metabolic pathways and related physiological processes. Since many genes are involved in the regulation of salinity tolerance, traditional approaches that examine one or a few genes in response to salinity may fail to capture and characterize the complex responses at the molecular level. Thus, for such quantitative traits, identifying functional gene clusters would be much more meaningful than searching for a single gene. With the advent of next‐generation sequencing technology, transcriptional responses to an environmental stimuli can be examined at a genome‐wide level and provide a comprehensive understanding of the complex processes underlying environmental adaptation and abiotic stress responses.

RNA‐sequencing data provide valuable information on gene expression across different experimental conditions, time points, tissues, or genotypes. Traditionally, in co‐expression network analysis, genes with similar expression pattern are grouped, with the underlying rationale being “guilt by association.” This extensively validated principle states that transcriptionally coordinated genes are often functionally related. Once co‐expression modules are identified, it is challenging to determine which modules are associated with the phenotypic response, and which biological processes in the same module are involved. To link modules to phenotype, one approach is to calculate the correlation between physiological traits and eigengenes of the given modules, which are defined as the first principal component (PC) of a specific module (Virlouvet et al., 2018). The first PC accounts for the largest variance of the gene expression for genes within the module and thus can describe the major expression pattern. This method is reasonable when the major variation in the data is caused by a treatment or condition. However, in practice, genes in the same module are not necessarily in the biological process due to different locations of gene products in cells, and mathematically, module‐discovery methods may introduce large variance in the clustering process. The correlation approach based on single average patterns may fail to identify modules associated with the trait.

To reveal the substructure of modules and identify submodules that are associated with the observed trait, principal component analysis (PCA) was used to break down modules and multivariate regression analysis was used to test the most significant submodules. Specifically, the variable selection method least absolute shrinkage and selection operator (LASSO) was employed to identify the substructure of gene modules and find the clusters of genes highly relevant to salt stress response in rice. Although various algorithms were developed for variable selection, LASSO is well known for its statistical accuracy, computational feasibility, and broad applicability to adaptation. In this work, we applied LASSO to the gene co‐expression network of rice with salt stress to discover key genes and their interactions for salt tolerance‐related phenotypes. LASSO‐based methods were applied to different biological research before. For example, it has been used GWAS analysis (Wu, Chen, Hastie, Sobel, & Lange, 2009), eQTL analysis (Cheng, Zhang, Guo, Shi, & Wang, 2014), transcriptome assembly (Li, Feng, & Jiang, 2011), and gene regulatory network analysis (Gustafsson, Hornquist, & Lombardi, 2005). However, it is the first application of LASSO method for the identification of submodules in gene co‐expression networks in plants.

## 2 METHOD AND MATERIALS

### 2.1 Plant growth conditions and phenotyping

All phenotypic data were collected from large‐scale phenotyping of a diverse panel of rice varieties. The greenhouse conditions and experimental description for these experiments can be found in the reference (Campbell et al., 2017). Briefly, the study used 383 of the 421 original RDP1 accessions and seven check varieties (Zhao et al., 2011; Famoso et al., 2011, Eizenga et al., 2014). According to the classification by Famoso et al., the subset of RDP1 included 77 *indica*, 52 *aus*, 92 *temperate japonica*, 85 *tropical japonica*, 12 *groupV/aromatic*, and 56 highly admixed accessions (the subpopulation assignment was not provided for nine accessions) (Famoso et al., 2011). The phenotyping experiments were conducted between July and September in 2013 in a controlled greenhouse at Lincoln, NE. The greenhouse was maintained at 25–28°C with relative humidity at 50%–80%, and a photoperiod of 16 hr:8 hr (day:night). Seedlings were germinated in the dark for 2 days, exposed to light for 12 hr, and were transplanted into pots filled with Turface (Profile Products, LLC). The seedlings were grown in tap water for 4 days after transplanting and were supplemented with half‐strength Yoshida solution (pH 5.8) for the remainder of the experiment. For salt treatment, NaCl was mixed with CaCl_2_ in a 6:1 molar ratio and was added after 10 days of seedling growth. The stress treatment was started at 2.5 dS/m and was increased gradually up to 9.5 dS/m in four steps over a period of 4 days. The stress treatment was maintained at 9.5 dS/m for the remaining 2 weeks. Root and shoot samples were collected separately and rinsed 3 times in tap water and once in deionized water to remove excess NaCl after the experiment (14 days of 9.5 dS/m; 28 days after transplant). The samples were oven‐dried at 60°C for 1 week prior to measuring root and shoot biomass. Shoot and roots from two plants were taken for biomass measurement. Dried shoot samples were ground and 200–300 g of total material was digested with 0.1 N Nitric acid (Fisher Scientific) at 70°C for 8 hrs, while root samples were weighed and digested without any grinding. The samples were diluted, and cation (Na^+^ and K^+^) concentrations were determined with an appropriate standard by dual‐flame photometry (Cole Parmer). Phenotypic data were combined across periods, and a linear model was fit to calculate adjusted means for individual accession using the PROC GLM procedure of the Statistical Analysis System (SAS Institute Inc). The linear model included a period (i.e., June–July or August–September), replication nested within a period, tub nested within replication, accession, and accession‐by‐period interaction.

### 2.2 Transcriptome experiment and RNA‐sequencing

RNA‐seq data were generated from shoot tissues of 92 diverse rice accessions. These accessions were randomly selected from the Rice Diversity Panel 1 (Zhao et al., 2011) and consist of 34 subspecies *Indica,* while 52 accessions were from subspecies *Japonica*. For each accession, gene expression profiles of shoot tissues were measured for both control condition and salt condition after exposing the rice seedlings to 6 dS/m (~60 mM NaCl) salt stress for 24 hr. The RNA‐seq data can be accessed through GEO database (Accession #: GSE98455).

### 2.3 RNA‐seq data analysis and Co‐expression network analysis

By using Trimmomatic (Bolger, Lohse, & Usadel, 2014), each 101 bp RNA‐seq read was trimmed to make sure the average quality score larger than 25 and having the minimum length of 75 bp. All trimmed short reads were mapped to the *rice* Genome (version 6) using TopHat (Trapnell, Pachter, & Salzberg, 2009), allowing up to two base mismatches per read. Reads mapped to multiple locations were discarded. Numbers of reads in genes were counted by the HTSeq‐count tool using corresponding rice gene annotations (Anders, 2010). DEseq (Anders & Huber, 2010) was used to do normalization for read counts of all genes.

Co‐expression network analysis was used to identify genes with coordinated transcriptional responses (modules). Genes exhibiting low variance or low expression across both control and salt samples were removed, as these genes could introduce noise with the co‐expression pattern measured with Pearson correlation. Two criterions were used for this purpose: (a) the ratio of upper quantile to lower quantile of normalized read count smaller than 1.5; (b) for more than 80% samples, normalized read count smaller than 10. To capture the signal of changes caused by salinity stress, a log2 fold change matrix was calculated by dividing the salt count with corresponding control count and further stabilized through log transformation. For this log2 fold change matrix used for co‐expression network construction, genes with the ratio of upper quantile to lower quantile larger than 0.25 were kept. Among the total of 57,840 *rice* genes, 8,953 genes displaying sufficiently high variation were identified, and their values were used to construct a correlation matrix using the R package, WGCNA (Langfelder & Horvath, 2008). The soft threshold was set as 4 to ensure the scale‐free topology to be higher than 0.9. Due to the complexity of the hierarchical clustering tree, method dynamic hybrid cut was implemented to get modules. Dynamic tree cutting was adopted to identify modules with minModuleSize of 25 (Langfelder & Horvath, 2007).

### 2.4 Algorithm for linking phenotyping data to submodules in gene co‐expression network

**Figure 1:**
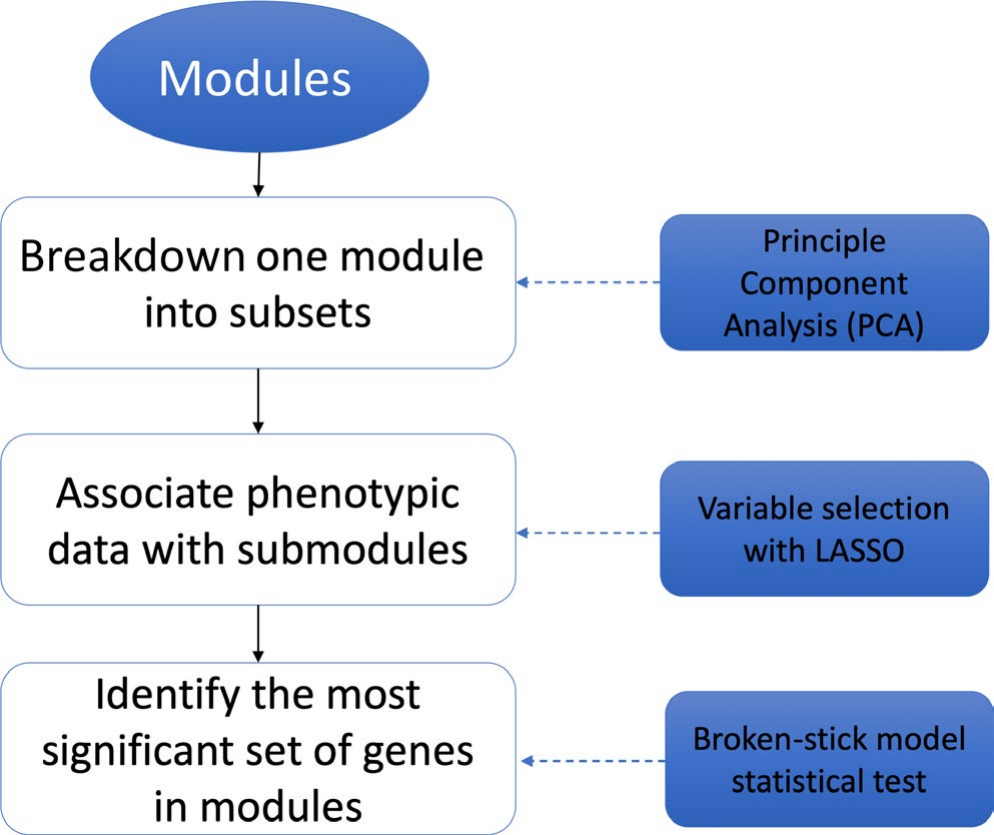
Flowchart of the algorithm to link phenotyping data to submodules in the gene co‐expression network

Figure 1 shows the workflow of the algorithm to link phenotyping data to submodules in the gene co‐expression network. For all modules identified by WGCNA, the first step is breaking down all modules into submodules. PCA was used for all modules. The first, second, and third components were considered, and the eigenvectors of the first three PCs were used as the virtual genes to represent genes in these components. Then, LASSO method was employed to select the most significant virtual genes associated with phenotyping data. The following section describes the details of the LASSO step. Once significant virtual genes identified, all genes in the same module were compared with a significant virtual gene to identify the most correlated genes with a statistical test based on the broken‐stick model. The details of this test are described in the following sections.

### 2.5 Variable selection with LASSO

Various algorithms were developed for variable selection, but LASSO is well known for its statistical accuracy, computational feasibility, and broad applicability to adaptation. In this manuscript, we applied LASSO to the gene co‐expression network of rice with salt stress. To link the phenotypic data to gene expression profiles, a linear model was fitted:

(1) $$Y_{i}=X_{i}\beta_{i}+\beta_{0i}+e,$$

where *Y*_*i*_ are the phenotypic response for the *i* ^*th*^ (*i *=* *1…92) genotype, *X*_*i(jk)*_ is the PC matrix that *X*_*i(jk)*_ represents the log_2_ PC value from the *j* ^*th*^ (*j *=* *1…3) PC of the *k* ^*th*^ (*k *=* *0…16) module for the *i* ^*th*^ genotype, and is the coefficient of the *j* ^*th*^ PC from *k* ^*th*^ module and its absolute value quantifies the contribution effects. The phenotypic response, *for example,* the physiological vector, was log_2_ of Na^+^/K^+^ ratio. The LASSO method was used to shrink coefficients of virtual genes with trivial effects into zeroes while keeping virtual genes with large effects by minimizing the residual sum of squares with an additional L1 norm, shown in Equation :

(2) $$\min\sum {\left(Y_{i}-\beta_{0i}-X\beta_{i}\right)}^{2}+\lambda \left[\left(1-\alpha \right)\left||\beta_{i}{\left|\right|}_{2}^{2}+\alpha |\right|\beta_{i}{\left|\right|}_{1}^{2}\right].$$

The optimal penalty parameter λ is a constant larger than zero, and the optimum value was determined with leave‐one‐out cross‐validation. To determine the optimal set of parameters selected by LASSO, we adopted the most regularized model such that error is within one standard error of the minimum.

### 2.6 Identification of significant genes with broken‐stick model

After significant PCs selected by the linear regression, we developed a broken‐stick model to identify genes significantly associated with the selected PCs. In stick‐breaking theory, a stick of length one would be literately broken into pieces and the length of broken pieces just follow the Dirichlet distribution. Here, we take the contribution values of genes from the same module as the lengths of pieces from a broken stick. The random sampling from the Dirichlet distribution was repeated for many times, and for each time, the broken pieces were sorted by their lengths in a descending order. The gene with the largest contribution would be compared with the upper quantile of the empirical distribution constructed by the largest lengths of broken pieces. If the contribution value is larger than the upper quantile from the random background, this gene would be regarded as genes that have an unusual contribution to the selected PC. For a module with *K* genes, a stick, whose length is unit 1,  needs to be broken into *K* pieces. The lengths of those *K* pieces were got from the following the Dirichlet distribution. We denote the length of the *i* ^th^ (0 < *i* < *K*) piece as *x*_*i*_ (0 < *x*_*i*_ < 1) and, therefore,  $\sum_{i=1}^{K}x_{i}=1$. In addition, for each *x*_*i*_, we have the corresponding α_i_ (α_i_ > 0). Then, random variables *X = (X_1_, X*_*2*_ *,…, X*_*k*_) have the following PMF (Equation ):

(3) $$f\left(X,\alpha \right)=\frac{\Gamma \left(\sum_{i=1}^{K}\alpha_{i}\right)}{\Pi_{i=i}^{K}\Gamma \left(\alpha_{i}\right)}\Pi_{i=1}^{K}{\left(x_{i}\right)}^{\alpha_{i}-1},$$

In our case, to make sure that *X* follows uniform distribution in the *k* ^*th*^ dimension, α_i_ (0 < *i* < *K*) was set as one. The sampling process was repeated for 10 thousand times, and for each time, the resulting lengths were further sorted in the descending order *x*_*(1)*_ *< x*_*(2)*_ *< … < p*_*(m)*_ *< … < x*_*(k)*_. Values of *x*_*(m)*_ from 10 thousand simulations would be used to construct the corresponding empirical distribution *E*_*(m)*_. Meanwhile, the proportions of contribution denoted as *p*_*(m)*_ *,* of genes in the module were sorted in the descending order *p*_*(1)*_ *< p*_*(2)*_ *< …p*_*(m)*_ *< … < p*_*(k)*_. The value of *p*_*(m)*_ was then compared with upper quantile of *E*_*(m)*_.

### 2.7 Real data‐driven simulation

Two different types of simulations were conducted to compare LASSO and correlation in selecting expression patterns.

### 2.8 Simulation I

In simulation I, LASSO method and a simple method based on the correlation were compared to test which one has a better performance in selecting the true PC patterns. A real data‐driven simulation was performed to evaluate whether LASSO is better in picking up correct expression patterns, *for example,* PCs, than the simple correlation comparison. In the simulation, the real PC matrix containing 51 PCs from 17 gene modules was used, and the same 8 PCs selected by LASSO with real data were assumed to be positives to contribute to the observed change in Na+/K+ ratio. The absolute value of their coefficients estimated by the linear regression without penalty is called effect sizes on the dependent variable. For the real case, the values of effect sizes are in the range of .034 to .1596. The comparison between LASSO and the correlation method was conducted by changing effect sizes obtained by multiplying the original coefficients of those 8 PCs with a series of multiplying factors ranging from .3 to 2. For other PCs not chosen, their coefficients were set as zeroes. Equation  describes the formula used in the simulation to calculate the dependent variables with the real PC matrix and predefined coefficients of all PCs.

(4) $$Y_{\left(N\times 1\right)}^{\mathrm{sim}}=X_{\left(N\times M\right)}\beta_{\left(M\times 1\right)}^{\mathrm{sim}}+\varepsilon_{\left(N\times 1\right)}^{\mathrm{sim}},$$

where *X*_*N*×*M*_ is the same PC matrix as what we used in real data analysis. $\beta_{{}_{M\times 1}}^{\mathrm{sim}}$ is the assumed coefficient for all PCs. The residual error $\varepsilon_{N\times 1}^{\mathrm{sim}}$ follows a normal distribution *N*(0, σ^2^), where the variance σ^2^ was estimated with the residual values from the linear regression with eight PCs. With the formula above, for each multiplying factor, we generated $Y_{N\times 1}^{\mathrm{sim}}$ for 100 times using different simulation seed. For each round of simulation, the ability of LASSO in identifying correct the PC pattern was compared with that of correlation method. Due to the skewed dataset that the number of true negatives dwarf the number of true positives, the area under precision‐recall curve (PR AUC) is used as the standard of comparison. The ranking of PC patterns for calculating PR AUC is based on the absolute values of the correlation between PC patterns and the simulated *Y*. For LASSO, the ranking is obtained from the Coefficient Shrinkage curve, in which coefficients of PCs would shrink to zeroes in order. If the shrinkage curves of PCs are shrunk to zero at the same time, they are further ranked by the absolute values of their coefficients at the optimum lambda.

### 2.9 Simulation II

We randomly choose eight PCs and set their coefficients as non‐zero values so that four of them had the same positive number and the other four had the same negative number. The maximum coefficient size from the real data analysis is .1596, and the minimum size is .034. Based on the scale of the original coefficients, coefficient series in our simulation is .03, .05, .1, .15, .3, and .5. For each effect size, we did 100 simulations. However, PCs set to have a non‐zero effect size are the same as what we picked from real data analysis. Moreover, the signs of their coefficients are unchanged, and their effect sizes are either decreased or increased in the same proportion. To make our conclusion more robust, eight PCs were randomly chosen out of the 51 PCs. The absolute values of their coefficients were set the same and four of them were assumed to have positive effects, while the other four were assumed to have negative effects. Based on the effect size in real data analysis, we tested a series of effect size, .03, .05, .08, .1, .15, .3, and .5. For each effect size, we repeat for 100 times with different seeds.

### 2.10 GO term enrichment analysis

GO::TermFinder (Boyle et al., 2004) was used to identify modules significantly enriched by genes belonging to GO terms. The *p* value was calculated with hypergeometric distribution and further adjusted with Bonferroni to correct multiple hypothesis testing. The cutoff used is adjusted *p* value < .05. The GO term association files for rice were obtained from http://rice.plantbiology.msu.edu/.

## 3 RESULTS

### 3.1 Phenotypic data and gene co‐expression network in response to salinity stress

**Figure 2:**
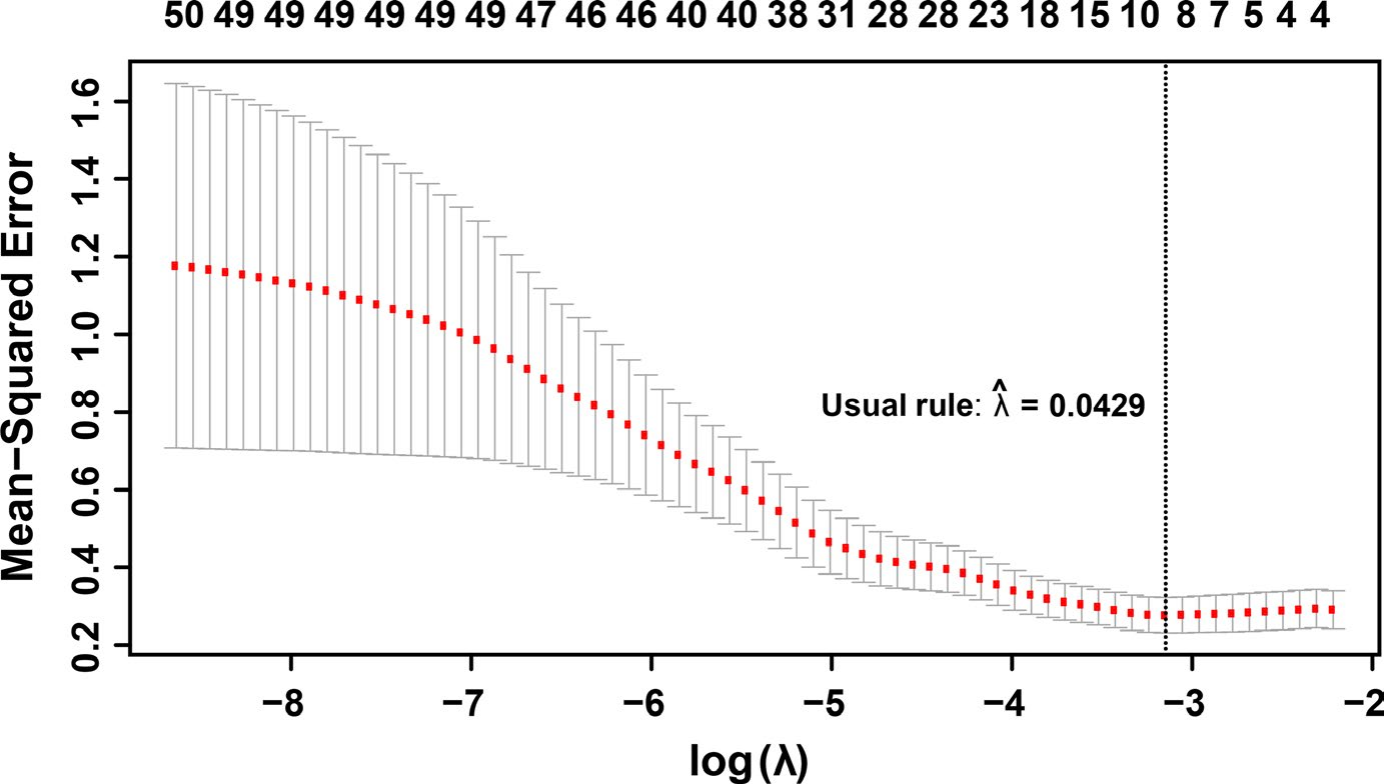
The clustering result of WGCNA to the gene co‐expression network with a heatmap plot. The heatmap shows the topological overlap matrix among all genes in different clusters, and blocks of darker colors along the diagonal are related to genes from the same modules

For this study, the primary aim was to identify genes or gene clusters whose expression patterns were highly associated with physiological responses to salinity stress. After a 9 dS/m (~90 mM NaCl) salt stress was imposed gradually over 4 days (in four increments of 20–30 mM) to 2‐week‐old rice seedlings, tolerance‐associated traits in rice, such as shoot biomass and shoot Na+ content, were measured at the end of a 2‐week stress period. In this study, the shoot Na^+^ content was used to represent the plant response to the salt stress. The inherent differences in growth rate between lines were controlled, and hence, the saline‐induced growth response was normalized by corresponding parameters in control conditions. To identify the gene clusters responding to salinity stress, a co‐expression network was constructed, in which genes are referred to as nodes and an edge between two nodes indicates that the corresponding two genes have similar expression patterns. The expression profiles used to construct the gene co‐expression network come from RNA‐sequencing data of shoot tissues across 92 diverse rice accessions. We performed the weighted gene co‐expression network analysis (WGCNA) on 8953 genes just for those 184 samples exposed to salt stress, and the clustering result is shown in Figure 2. Please see the Section of Material and Methods for more details. All those genes were distributed into 17 modules, with the size ranging from 34 to 2,963 genes. These modules and the shoot Na^+^ content were integrated with a linear model to link transcriptomic changes to rice phenotypic response to salinity stress.

### 3.2 Module features selected by LASSO

**Figure 3:**
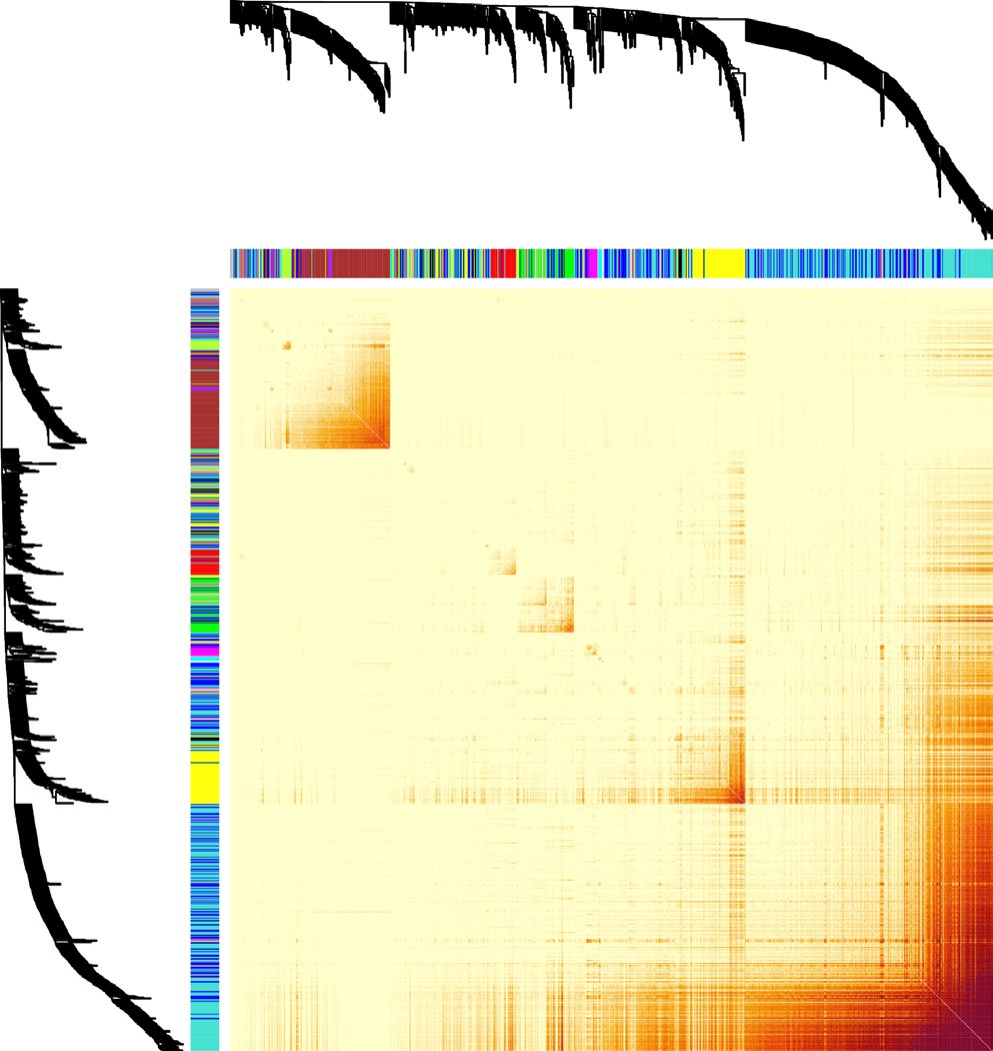
For LASSO training result, the cross‐validation errors were plotted against varying log(λ) values in the search range

**Figure 4:**
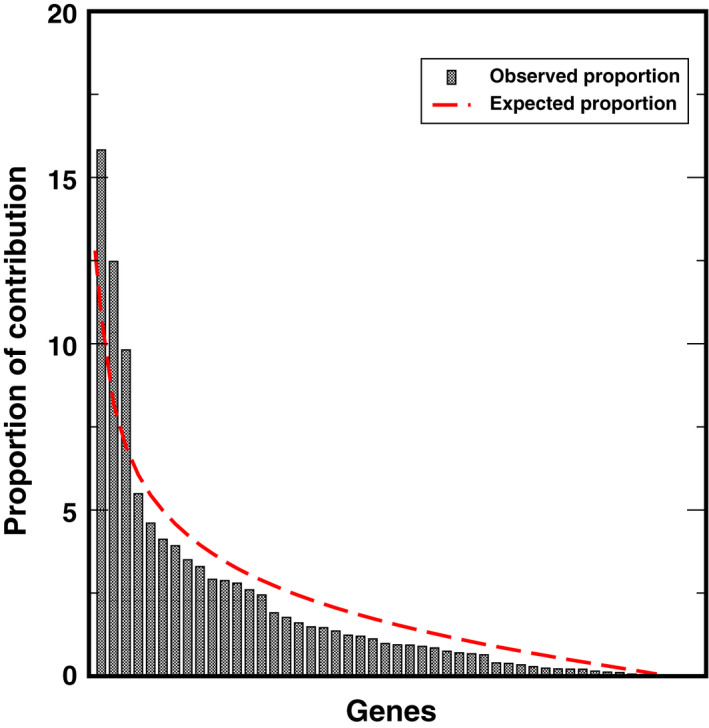
The contribution of genes to PC2 in Module #14 with the background

**Figure 5:**
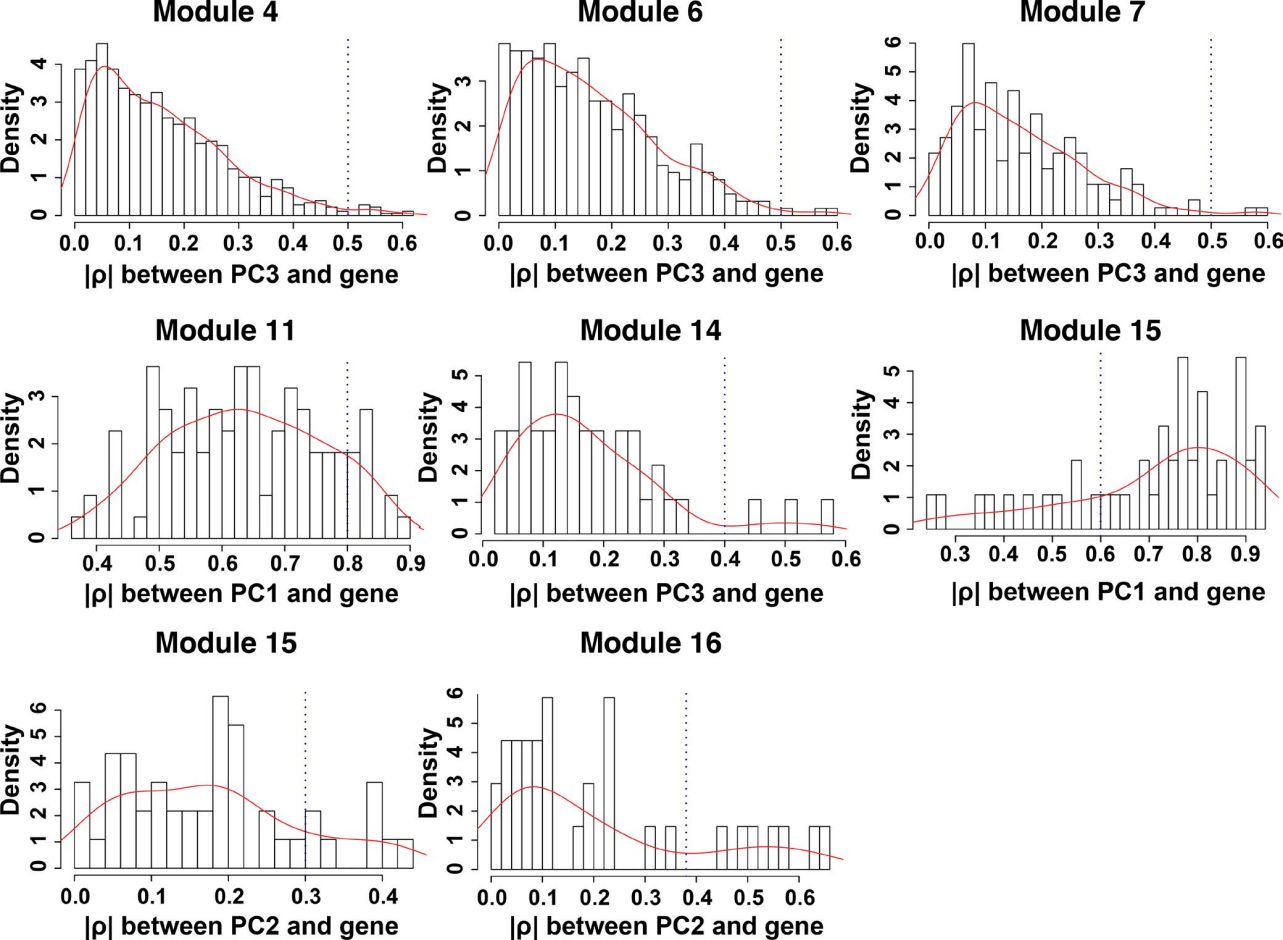
The distributions of genes with respect to the correlation to each specific PCs.

Once co‐expression modules are identified, we next sought to identify modules that are related to salinity stress. Traditionally, PCA would be performed on gene expression profiles of each module to get the first PC of each module (also called the eigengene), and the importance of each module was evaluated by the strength of correlation between eigengenes and the physiological trait (Virlouvet et al., 2018). However, genes in a module identified in the co‐expression network are heterogeneous and could be involved in many different biological processes and respond to variant signals. Therefore, we hypothesized that one module has substructures, and a submodule responds to a specific signal. A new method to select submodules associated with the trait of interest (i.e., shoot Na^+^ content) was developed. In the first step, PCA was performed on each module and extracted the top three PCs of each module to form a PC matrix (total of 51 PCs from 17 modules). Only top three PCs were taken from each module because the higher‐order PCs have a very low contribution to the entire module and hence can give rise of an overwhelming noise level. One can find if display genes in the eigengene space, such as PC1 and PC2, genes can be grouped into different clusters: Some genes are close to PC1 and the other to PC2. This indicates that genes in the same module still can be further split into submodules. The second step is the statistical feature selection step—a regularized regression model, a LASSO‐based method, was applied to quantify the relationship between module expression patterns and the physiological data. The fitted model can find the expression patterns contributing the most to the observed physiological data. During the PC selection step, using LASSO, the optimal λ values were identified with a leave‐one‐out cross‐validation. The result is shown in Figure 3, where the cross‐validation errors were plotted against varying log(λ) values in the search range. The error bars show the standard deviation of the errors calculated from the cross‐validation. The dotted line indicates the λ giving the minimum mean squared error, and the corresponding value of the parameter is .0429. Therefore, eight PCs were identified as the optimal feature set. In other words, eight significant PCs from seven modules were selected to have non‐zero effects on the stress (Table 1). Interestingly, for most modules, the selected PCs are the second (module 15, 16) or the third PC (module 4, 6, 7, 14, 15, and 16), which would be missing by traditional methods using the first PC only. This result is reasonable because genes contributing to the first PCs are expressed for the maintenance of basic cellular functions, and genes’ expression for the response to environmental perturbation is a small part of the entire transcriptome. The last step is to identify genes significantly associated with the selected PCs. This step was implemented with a statistical test based on the broken‐stick model. In on gene module, the contribution of genes to a PC is considered as the lengths of broken pieces from a stick. Sorted contributions of genes would be compared with the upper quantile of the empirical distribution constructed by the largest lengths of broken pieces. If the contribution value of a given gene is larger than the upper quantile from the random background, this gene would be regarded as genes that have an unusual contribution to the selected PC. Figure 4 shows the comparison for genes to PC3 in Module #14, and three genes have significant combustion to PC3. The numbers of significant genes for nine PCs from eight modules are listed in Table 2. The distributions of genes with respect to the correlation to each specific PCs are shown in Figure 5. One may note that selected genes for a second or third PC in each module form a small peak before a large peak, which further indicates there are substructures in modules. For second and third CPs, the selected genes are a small portion of the entire module, and these genes have high potential to respond to the stress.

**Table 1:** Significant submodules after LASSO selection based on their coefficient values

Module #	4	6	7	11	14	15	16
1st PC				−.0325		−.0111	
2nd PC						.0862	.0869
3rd PC	−.0275	.01257	.0463		.0889		

**Table 2:** Overview of all significant modules

Module #‐PC	No. of genes in modules	No. of genes in submodules	Enriched with genes belongings GO terms	Adj. *p*‐value
4‐3	891	67	Transport (20/67)	1.9 × 10^−5^
6‐3	313	53	Response to stress (16/53)	1.58 × 10^−5^
7‐3	184	24		
11‐1	110	110	Response to stress	7.86 × 10^−9^
14‐3	46	3		
15‐1	46	18	Response to abiotic stimulus	.0089
15‐2	46	8		
16‐2	34	7	Cellular homeostasis (3/7)	4.46 × 10^−12^

### 3.3 Significant genes selected by LASSO are relevant to salt stress

All selected significant submodules are enriched with genes in GO terms relevant to salt stress (Table 2). Some of them are genes responding to stress and stimulus. For example, sixteen genes respond to the 3rd PC in **Module #6** are enriched by genes belonging to the GO term of “Response to Stress.” In this submodule, several genes encode transcription factors in the WRKY family, and overexpression of these genes resulted in enhanced salt and drought tolerance, in addition to increased disease resistance (Jiang & Deyholos, 2009; Ma et al., 2017; Qiu & Yu, 2009) or improves the osmotic stress tolerance (Song, Jing, & Yu, 2009). WRKY genes were also identified to respond to stress by an eQTL method in barley (Wehner, Balko, Humbeck, Zyprian, & Ordon, 2016). Some submodules are directly related to salinity conditions. For example, 20 genes associated with the third PC in **Module #4** are enriched with genes in “Transport” (*p*‐value = 1.9 × 10^−5^). For example, gene *LOC_Os01g37690* encodes a protein in NCX family and this protein also has a CAX domain H+/Ca^2+^ exchanging. The sodium/calcium exchanger protein that can maintain cellular homeostasis of Ca^2+^ or Na+. While one calcium ion is pumped outside of the cell, three sodium ions would be transported into the cell in exchange. This process could work in another direction depending on the concentration gradient of ions (Yu & Choi, 1997). Its homolog gene in *Arabidopsis*,* AtNCL*, is broadly expressed in *Arabidopsis*, and abiotic stresses stimulated its transcript expression. Loss‐of‐function *AtNCL* mutants were less sensitive to salt stress than wild‐type or transgenic overexpression lines (Wang et al., 2012). Another gene, *LOC_Os12g07270*, encodes a protein of BASS2, which is responsible for pyruvic acid uptake into the chloroplast, an essential precursor of ABA. It has been proved that a pyruvate transporter, TaBASS2, positively regulates salinity tolerance in wheat (Zhao, Ai, Wang, Xiao, & Xia, 2016). Other submodules, such as in Module #16, #7, #15, are also enriched with genes specifically responding to the salinity conditions.

**Module #16** has a total of 34 genes, and only seven genes are significantly aligned into the subgroup represented by the second PC. Three out of seven genes in this submodule have clues about their functions, and interestingly, these functions are highly relevant to salt stress. *LOC_Os12g01530 and LOC_Os11g01530*, two ferritin homologs, are function‐unknown genes in rice, but their homologs in other plants have functions to store ferrous iron in chloroplasts in a non‐toxic form and to protect plants from oxidative damage induced by different stresses, including salt stress (Deak et al., 1999; Foyer, Lelandais, & Kunert, 1994). Especially, with salinity stress, rice highly prone to have iron deficiency due to a lower release of Fe‐chelating compounds (Abbas, 2015). The correlation between expression levels of these two genes and Na^+^ concentration in shoot gives rise to a hypothesis that upregulated ferritin in salt‐tolerant rice helps the plant to survive under the salinity condition. *LOC_Os09g23300*, the third gene in this submodule, codifies a vacuolar iron transporter and also responds to salt stress. It has been reported that both *LOC_Os12g01530/LOC_Os11g01530*, encoding iron storage proteins, and *LOC_Os09g23300*, encoding one putative vacuolar iron transporter, are upregulated in shoot tissue caused by the stress of phosphate derivation (Secco et al., 2013).

In **Module #7**, there are 184 genes, but 24 genes consist of the submodule represented by the 3^rd^ PC. Out of 24 genes, the most interesting gene is *LOC_Os07g19030*, which can encode a tic22‐like family domain‐containing protein. Tic22, translocon at the inner envelope membrane of chloroplasts, is majorly involved in protein precursor import into chloroplasts (Kessler & Schnell, 2009). It has been reported that this protein can be induced and accumulated in salt‐acclimated cells in *Synechocystis* sp. strain PCC 6803 (Fulda et al., 2006). *LOC_Os10g30540* is a putative lectin‐like receptor kinase (LecRLK), which is well known for its role in plant stress and developmental pathways. For example, in *Arabidopsis,* LecRLK can respond to salt within the ethylene signaling pathway (He, Zhang, Yan, Zhang, & Chen, 2004). LecRLK in pea plant, being shown to phosphorylate MBP, has a unique response to Na+, and the transcript of the LecRLK accumulates in roots and shoots with salt stress (Joshi, Dang, Vaid, & Tuteja, 2010). *LOC_Os07g14100* is a gene coding a polygalacturonase (PG), one of the hydrolases responsible for cell wall pectin degradation, which is involved in organ consenescence and biotic stress in plants. In rice, the transcription of PG is induced by cold, salinity, and drought stresses, as well as by abscisic acid (ABA) treatment, and overexpression of PG can enhance sensitivity to cold, salinity, and drought stresses (Liu et al., 2014). Reduced violaxanthin de‐epoxidase, the gene product of *LOC_Os04g31040*, is instrumental in the regulation of the xanthophyll cycle, which can reduce reactive oxygen species (ROS) damage to cell structure during salinity stresses (Borah et al., 2017; Latowski, Kuczynska, & Strzalka, 2011).

**Module #15** has a total of 46 genes and both PC1 and PC2 are significant. Genes in these two submodules have functions to respond to stress. For example, *LOC_Os10g16974* and *LOC_Os10g17260*, genes codifying for cytochrome P450, are involved in growth and drought stress responses in rice (Tamiru et al., 2015). The gene product of *LOC_Os02g14680* is a UDP‐glucuronosyl and UDP‐glucosyl transferase domain‐containing protein and that of *LOC_Os01g71670* is a glycosyl hydrolase. Both genes are related to glcosylation. It is known that glycosylation is important for plants to respond to stresses; manipulation of glycosylation alters tolerance to biotic and abiotic stresses (Bowles, Isayenkova, Lim, & Poppenberger, 2005; Bowles, Lim, Poppenberger, & Vaistij, 2006). *LOC_Os10g38140* encodes a glutathione S‐transferase, by which the salt stress‐induced lipid peroxidation is reduced (Katsuhara, Otsuka, & Ezaki, 2005). *LOC_Os11g30500* is an HVA22 protein gene. In Barley and *Arabidopsis*, aleurone cells transformed with HVA22 inhibited the formation of GA‐induced formation of vacuoles and programmed cell death (Guo & Ho, 2008). Since vacuoles are important for Na+ storage, HVA22 is a promising candidate protein for salt tolerance. For example, a homolog gene of HVA22 from barley, HVA1, can increase tolerance to water deficit and salt stress in transgenic rice (Xu et al., 1996).

## 4 DISCUSSION

### 4.1 Linking gene expression to phenotypic data

The gene co‐expression network models have been used for the exploration, interpretation, and visualization of the relationship among genes in a wide range of biological applications (Kadarmideen & Watson‐Haigh, 2012; Tan et al., 2017; Yang et al., 2014), but was not integrated with phenotyping data directly yet. The method describes in this manuscript provide an approach to link phenotyping data to transcriptomic data, which provide complementary integration to QTL, the linkage between phenotyping data and genomic data, and eQTL, the linkage between phenotyping data and transcriptomic data. Co‐expression network analysis was combined with eQTLs (Villa‐Vialaneix et al., 2013), studying gene‐phenotype association (Ficklin, Luo, & Feltus, 2010), and GWAS (Schaefer et al., 2018). The discovered gene submodules and genes in these submodules from the method described in the manuscript can also be further combined with eQTL, QTL, and/or GWAS to prioritize genes responding to stress.

### 4.2 Response to stress with multiple submodules

To link modules to phenotype, the naive way (Virlouvet et al., 2018) is calculating the correlation between physiological traits and eigengenes of given modules, which are defined as the first PC of a specific module, accounting for the largest variance of the gene expression within the module. However, in practice, genes in the same module are not necessarily in the same biological process due to different locations of gene products in cells, and mathematically, module‐discovery methods may introduce large variance in the clustering process. Therefore, the correlation approach based on single average patterns may fail to identify modules associated with the trait. Therefore, multiple PCs in on modules need to be considered, and more than one significant PCs can be selected by LASSO. On the other hand, to generate a given type of response to environments by organisms, many genes or various pathways need to work together. Multiple submodules associated with different PCs are integrated together by the linear model can quantitatively describe the different levels of contributions of genes in these submodules and pathways enriched by these genes in the biological systems. At a certain level, the weight parameters of submodules assigned by LASSO can reflect the size of their contributions to the entire system.

### 4.3 Variability for different PCs and high‐order PCs

We used the top three PCs to represent the different submodules in one given module because they already dominate the contributions from all PCs. PCA was performed on all 17 modules. The first PC of each module accounts for 35%~62% of the total variation in gene expression, and the top three PCs could explain 42%~70% of the module variance. Some modules have less variation, that is, PC1 contributing a high percentage to the total variation, but the other modules have more variations. The total variation in gene expression could come from the response to the environmental perturbation, but also comes from the genetic population and even experimental artifacts.

### 4.4 Simulation study

A real data‐driven simulation was used to evaluate whether or not LASSO has a better performance in terms of picking up all correct expression patterns, a specific set of PCs, when compared with direct selection based on correlation. The details of the algorithm about the simulation are described in the section of Method.

**Figure 6:**
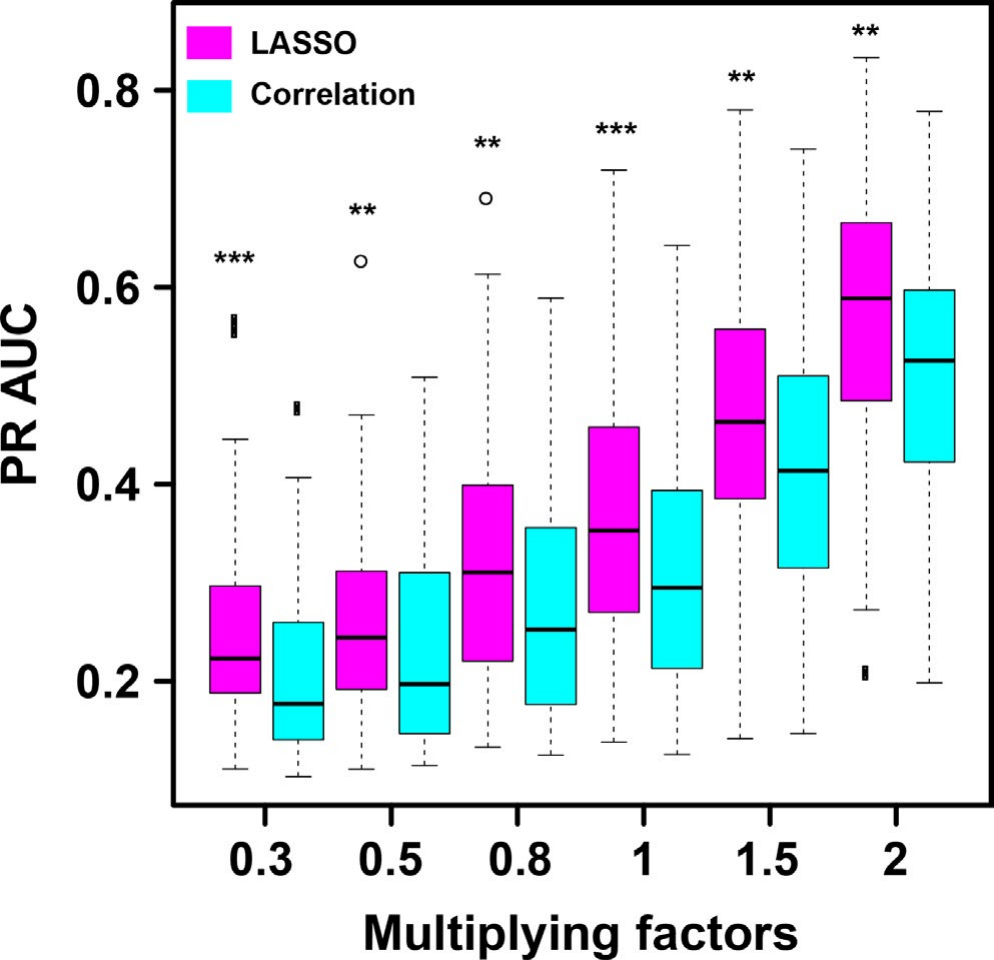
Simulation results of PR AUC comparison between LASSO and correlation method**.** The *x*‐axis represents the different multiplying factors. The box plot displays the 25th and 75th percentiles around the median value. Magenta box stands for LASSO method, whereas the cyan box represents the correlation method. The significance was calculated with Wilcoxon signed‐ranks test and *p* < .05 is labeled as *, *p* < .01 is labeled as **, and *p* < .001 is ***

From Figure 6, one can see that, as the effect size increases, the PR AUC values increases for both LASSO method and correlation method. (Please see the section of Method for the definition of the effect size.) It is suggested from the simulation result that PCs with larger coefficients have higher possibilities to be the true patterns associated with the phenotype. Although the PR AUC values are not high, the power of the LASSO method is significantly higher than that of correlation method. In practical research, biological annotation and GO annotation could further help remove those false positives.

**Figure 7:**
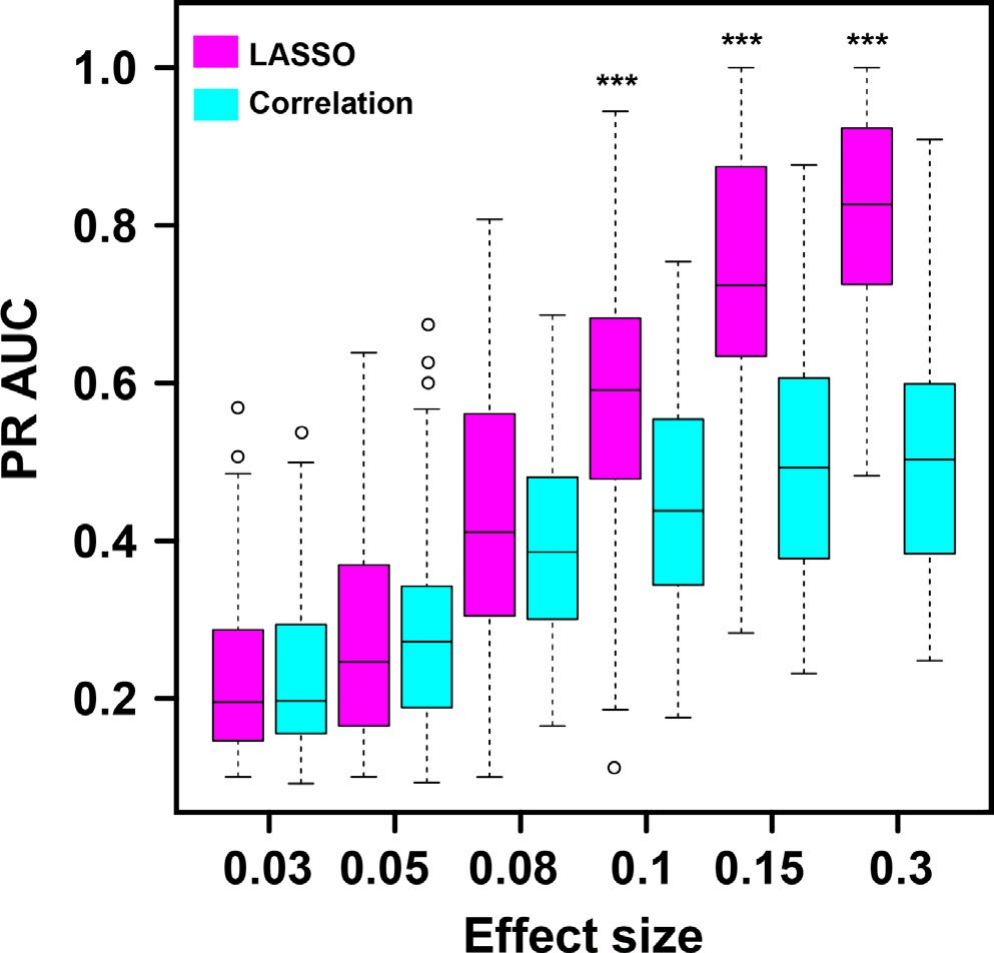
Simulation results of PR AUC comparison between LASSO and correlation method. The *x*‐axis represents different effect size. The box plot displays the 25th and 75th percentiles around the median value. Magenta box stands for LASSO method, whereas the cyan box represents the correlation method. The significance was calculated with Wilcoxon signed‐ranks test and *p* < .05 is labeled as *, *p* < .01 is labeled as **, and *p* < .001 is ***

From Figure 7, one can see that, as the effect size increases, the PR AUC values for both LASSO method and correlation method increase. A sharp increase could be observed when the effect size reaches .08. When we have low effect size, like .03, .05, and .08, there is no difference between LASSO method and correlation method. That means, for modules with trivial effect on the phenotype, we might do not have enough power to detect them even with LASSO method. The advantage of LASSO method begins to show up when the effect size climbs to 0.1. Also, the PR AUC values could reach around 0.8, which endows us with more confidence.

## 5 CONCLUSION

To link gene co‐expression network to stress phenotype data, a linear model based on LASSO method was applied to the gene co‐expression network of rice with salt stress to discover key genes and their interactions for salt tolerance‐related phenotypes. Submodules in gene modules were identified, and the linear relationship between these submodules and physiological responses of rice under salt stress was discovered. Genes in these submodules have functions related to ion transport, osmotic adjustment, and oxidative tolerance, which are biologically meaningful and useful for studies on rice salt tolerance. This method can be applied to other studies to efficiently and reliably integrate co‐expression network and phenotypic data, and also can be integrated with QTL, eQTL, and GWAS studies.

## CONFLICT OF INTEREST

The authors declare no conflict of interest associated with the work described in this manuscript.

## AUTHOR CONTRIBUTIONS

QD designed the study and implemented the algorithm. MC prepared the RNA‐seq data and phenotypic data. HY and KL helped the RNA‐seq data analysis and association study. QZ contributed to the statistical method and supervised the method development. CZ drafted the manuscript. CZ and HW supervised the whole project. All authors read and approve the final manuscript.
