# Supplementary material for: Network‐based feature selection reveals substructures of gene modules responding to salt stress in rice
Source: Plant Direct. 2019 Aug 12;3(8):e00154. doi: 10.1002/pld3.154 (PMC6689793; doi:10.1002/pld3.154)
Supplement: Supplementary file 1 [file PLD3-3-e00154-s001.pdf]

Point-to-point response

Re: 2019-00288
Network-based feature selection reveals substructures of gene modules responding to salt stress in rice

Dear Dr. Ana Fortes and Reviewers,
We are deeply grateful to your constructive comments, which have helped enormously to improve the quality of the manuscript. We have revised the manuscript accordingly. The modified parts are highlighted in yellow in the revised manuscript. The detailed point-by-point responses to the comments (*italic font*) are provided below. We hope that the revised manuscript is now acceptable for publishing

Thank you for your consideration!
Sincerely,

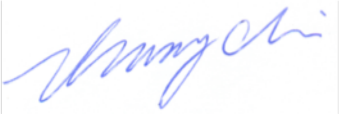

Chi Zhang

Response to the comments of Reviewer number 1

This manuscript has not been sufficiently edited to meet the minimum requirement for peer review. The article has no conclusion section, and the writing lacks sufficient spell-checking. The results section consists of one very short subsection, and one very long subsection which is very poorly organized and structured, and consists of a mash up of information belonging to methods, and discussion sections. Their proposed algorithm is badly described for a general audience, some of their mathematical formulas are not properly composed.

Response: We revised the manuscript carefully as per the advice of Reviewer #1. The manuscript was significantly revised and reorganized. A conclusion section was added, some mathematical formulas were corrected, and more discussions were edited.

Response to the comments of Reviewer number 2

This manuscript is an updated version of a study that was previously published without peer review at bioRvix: doi: https://doi.org/10.1101/359364.
To my knowledge, the manuscript describes the first application of the least absolute shrinkage and selection operator (LASSO) method for analysis of gene co-expression networks in plants. There are already many published reports and reviews on the development and application of this methodology in the biomedical field. Specifically, the current study expands the application of this method to plants by using RNA-seq data describing the response of shoot tissue of about 400 rice genotypes to salt (sodium) stress. It uses data simulation to compare results generated with conventional methods of correlation-based expression network identification to the expression modules generated with the regression-based network estimation. Finally, it uses gene-ontology analysis to infer the role of selected genes belonging to different modules (i.e. gene expression networks) in modulation plant's salt stress response, primarily as a measure of candidate gene prediction quality.
This study is of interest to a broad range of plant biologists as it illustrates the importance of data analysis method selection in RNAseq studies.
The paper is generally well written. It is very much a theoretical study. The discussion is limited to

methodological considerations and lacks connectivity to the applied aspect of this work, namely the use of the greatly improved gene-expression network prediction to identification of candidate genes for QTLs or eQTLs.

Response: The manuscript was revised accordingly, especially the discussion part. The connection of this method to QTLs and eQTLs was discussed in the new version.

I have the following more specific comments that should be considered to strengthen the manuscript:
1.The authors should cite reports on application of lasso-based methods in the biomedical field and should qualify statements on novel nature of the methodology developed by them.
2.The description of candidate genes and speculation about their possible role ins lengthy it could be at least partially replaced with a discussion how their findings relate of could be applied to the mapping of salt tolerance QTLs.

Response: We revised the manuscript according to the advice of Reviewer #2**.** We discussed applications of LASSSO in biomedical research and the potential connection of the mapping of salt tolerance QTLs.

Response to the comments of Reviewer number 3

In the work entitled "Network-based feature selection reveals substructures of gene modules responding to salt stress in rice" the authors applied the LASSO regression method in order to integrate gene co-expression network and stress phenotype data aiming to conduct a systems analysis to link genotype to phenotype. Such analysis was dome on rice under salt stress conditions.

Overall, the manuscript is organized properly and easy to follow the experiments and the hypothesis behind it. Pictures and graphs illustrate well the results and are described accordingly. The text requires some small grammar corrections.

Major reviews
- Genes must be written in Italic.

Response: We revised the manuscript accordingly, and all fonts were corrected**.**

Minor reviews
Line 264 - ..."to zero at the"...
Line 266 - ..."that four of"... something is missing after this.
Results
- Lines 374-375 - Please review the grammar.
- Line 377 - ..."gives rise to"...
- Line 393 - ..."is a gene of Polygalacturonase (PG)"... family?? Something is missing in this sentence.
- Line 404 - ..."genes codifying for... are involved..."
- Line 405 and thereafter - Please, don´t write: ..."is a gene for a given protein. Instead, write that the gene XXX codifies for....
- Lines 407-408 - Please review.

Response: The manuscript was revised carefully based on the above advice provided by Reviewer #3.

- A short description of plants growing under stress will introduce the reader to the phenotype.

Response: A brief description was added to introduce readers to the phenotype.

- From line 347, the authors always say that genes are enriched at a given GO terms. I have a problem with this. Usually, a module, cluster or category is enriched in genes belonging to a specific GO category. I don´t think genes are enriched. A group is enriched with genes responsible for salt stress response (for example). In line 366, the authors wrote it properly.

Response: In the revised version, we corrected all wrong parts about “enrichment”.

- <in the first section of results - Gene co-expression network in response to salinity stress - here, there is no figure illustrating the WGCNA analysis what would greatly help the reader to further follow the LASSO analysis. I find this very important.

Response: A new figure, Figure 2, was added to show the WGCNA result.
